# Prioritising referrals of individuals at-risk of RA: guidance based on results of a 10-year national primary care observational study

**DOI:** 10.1186/s13075-022-02717-w

**Published:** 2022-01-18

**Authors:** Leticia Garcia-Montoya, Jacqueline L. Nam, Laurence Duquenne, Catalina Villota-Eraso, Andrea Di Matteo, Collette Hartley, Kulveer Mankia, Paul Emery

**Affiliations:** 1 Leeds Institute of Rheumatic and Musculoskeletal Medicine, Chapel Allerton Hospital, University of Leeds, Leeds, LS7 4SA UK; 2 National Institute for Health Research Leeds Biomedical Research Centre, Leeds Teaching Hospitals NHS Trust, Leeds, UK; 3 Universidad de La Sabana, Department of Rheumatology, Chía, Colombia; 4 Rheumatology Unit, Department of Clinical and Molecular Sciences, “Carlo Urbani” Hospital, Polytechnic University of Marche, Jesi, Ancona, Italy

**Keywords:** Rheumatoid arthritis, Anti-CCP, ACPA, Autoantibodies, Epidemiology, Joint pain, Primary care, Risk, Inflammatory arthritis, Progression

## Abstract

**Background:**

Musculoskeletal (MSK) symptoms are among the commonest reasons for primary care assessments; however, few individuals will be diagnosed with an inflammatory arthritis (IA) within the following year. The purpose of this study was to investigate, in individuals with new MSK symptoms, the association between patient factors and risk of progression to IA, in order to optimise primary care referrals to rheumatology.

**Methods:**

Individuals ≥16 years old with new non-specific MSK symptoms and no clinical synovitis were recruited by primary care across the UK from July 2007 until May 2019. Those testing positive for the anti-CCP2 assay (anti-CCP+) were invited to Leeds for follow-up. Subjects with a negative result (anti-CCP−) were sent a 1-year questionnaire, and general practitioners were contacted to confirm whether the individual had been diagnosed with an IA by a rheumatologist. Predictors for progression were assessed using multivariable regression analysis.

**Results:**

Six thousand seven hundred eighty individuals were recruited: 3% were anti-CCP+, of whom 45% progressed to IA, predominantly rheumatoid arthritis. Anti-CCP+ participants with high antibody levels had an odds ratio (OR) for progression to IA of 9.42 [*P* < 0.001, 95% CI (3.13–28.30)], hand pain, OR 2.74 [*P* = 0.043, 95% CI (1.03–7.27)] and foot pain, OR 4.10 [*P* = 0.003, 95% CI (1.59–10.54)]. In low-level anti-CCP+ individuals, absence of pain in hands or feet had a negative predictive value of 96% for progression to IA.

One-year follow-up data were available for 5640 anti-CCP− individuals, of whom 53 were diagnosed with IA (0.93%). Pain in hands, OR 2.51 [*P* = 0.018, 95% CI (1.17–5.39)] or knees, OR 3.03 [*P* = 0.003, 95% CI (1.47–6.25)] were associated with development of IA within 12 months.

**Conclusions:**

This is the largest prospective primary care study of individuals at risk of IA, and the first one to prospectively investigate the outcome of MSK symptoms in a large anti-CCP− cohort. High anti-CCP levels and pain in hands/feet indicated an increased likelihood of progression to IA. In patients with low anti-CCP level and no pain in the hands/feet, progression is unlikely. In anti-CCP− patients, those with hand or knee pain were at increased risk of progression. This study demonstrates that routinely available tests and joint symptoms provide useful discrimination that may be used to prioritise referrals to rheumatology and avoid a delayed diagnosis.

**Trial registration:**

NCT, NCT02012764. Registered 25 January 2007.

**Supplementary Information:**

The online version contains supplementary material available at 10.1186/s13075-022-02717-w.

## Background

Early treatment of rheumatoid arthritis (RA) has demonstrated better long-term outcomes [1, 2]; however, there are limitations for guaranteeing prompt referrals to rheumatology services. Firstly, patients can experience joint pain for a long time before requesting an appointment with their general practitioner (GP). Secondly, when this appointment happens, the patient may not have any inflammatory symptoms, and therefore, they might have to see their GP several times before referral to a specialist is considered [3]. Thirdly, due to a shortage of rheumatologists, patients sometimes have to wait for several months to be assessed [4] at which point the disease might have become more established. Therefore, it would be helpful if primary care referrals to rheumatology could be prioritised according to the risk of progression to inflammatory arthritis (IA) [5].

Anti-cyclic citrullinated peptide (anti-CCP) antibodies are associated with progression to RA [6, 7], and they can be found in blood samples years before the development of clinical synovitis [8]. They are present in 1% of the general population [9, 10], but the progression rate of these individuals can be as low as 1% per annum [3]. This means that anti-CCP screening in the general population is not cost-effective, and other factors must be taken into consideration. For this reason, selecting individuals with new non-specific musculoskeletal (MSK) complaints could provide a cohort enriched for anti-CCP positive (anti-CCP+) individuals, with a higher risk of progression to IA [11].

Other primary care studies have focused on the overall pattern of joint pain, symptoms in key joints or the physical examination [12–14]; however, none have used patients’ symptoms to estimate the risk of progression to IA.

The main objective of this study was to determine, in anti-CCP+ (high and low level) and anti-CCP negative (anti-CCP−) individuals presenting to primary care with a new non-specific MSK complaint, the demographic features and patient-reported symptoms, which were associated with progression to IA. The ambition being to facilitate guidance in primary care regarding the risk of progression to IA, so that individuals likely to develop the disease can benefit from early referral to rheumatology services.

## Methods

This study analysed data from a prospective cohort of individuals from an observational study adopted by the National Institute of Health Research (NIHR), Clinical Research Network (CRN) and approved by the Leeds West Research Ethics. Participants gave written informed consent to take part in the study and were recruited from primary care centres across the UK from July 2007 until May 2019. To be eligible for the study, subjects had to be at least 16 years old and have a “new” non-specific MSK symptom that had not been previously reported to their GP. Clinical synovitis, current use of immunosuppressants, previous use of disease-modifying antirheumatic drugs (DMARDs) and a diagnosis of IA were exclusion criteria.

Most referrals were made by GPs; however, other healthcare professionals such as nurses, physiotherapists, and MSK physicians were also involved in recruitment. Participants were asked to fill in a questionnaire regarding any previous or current MSK diagnosis, family history of RA (and if so, who) and smoking status. They were also asked to mark their symptomatic joints on a diagram: neck, back, shoulders, elbows, wrists, hands, thumbs, hips, knees, ankles and feet.

Following this, a blood sample taken at their local GP practice was sent to Chapel Allerton Hospital (CAH) (Leeds) for analysis. A second generation anti-CCP assay was used to determine the presence of anti-CCP antibodies. Positivity of the test was determined using machine-specific cut-offs—initially using an ImmunoCAP 250 (Phadia) (reference range < 7 U/mL) and later on a BioPlex 2200 (Bio-Rad) machine (reference range < 2.99 U/mL). Three times the upper normal limit was considered high anti-CCP+ and below that low anti-CCP+ [15].

Individuals with a positive anti-CCP result were invited to attend a dedicated research clinic at CAH (Leeds) for further assessments. These participants were followed up in secondary care 3 monthly for a year and then annually until progression to IA. Progression to IA was determined by a rheumatologist and confirmed with an ultrasound (US) scan of the joints.

Subjects with a negative anti-CCP test received standard care by their GPs and were sent a postal questionnaire 12 months after enrolment asking about their disease status. Anti-CCP+ subjects unwilling to attend clinic also received standard care by their GPs and a 12-month postal questionnaire but in addition were contacted periodically by the team, either by telephone or by post to assess disease status. If any participant (anti-CCP+ or anti-CCP−) that did not attend clinic at CAH reported disease progression, GPs were contacted to confirm the participant status: only individuals whose GP confirmed that IA diagnosis had been made by a rheumatologist were considered progressors. Follow-up ended when the subject developed an IA.

### Statistical analysis

Statistical analyses were performed using SPSS version 21. The date of collection of the first blood sample was used as the baseline date. For analysis purposes, family history of RA was defined as a first-degree relative (FDR) diagnosed with the disease and this was coded as a dichotomous variable. Smoking status was also coded as a dichotomous variable (ever smoked: yes/no). Chi-square and *T*-test were used to assess relationship between two categorical and two continuous variables respectively. Association of the variables with the development of IA was done using binary logistic regression, first in a univariable model and later in a multivariable model. This latter model was adjusted for confounders: sex, age, family history of RA, smoking exposure and anti-CCP level. Multiple imputation was performed for missing data (5% missing smoking exposure and 5% missing family history of RA). In addition, for anti-CCP+ individuals, time for progression to IA based on the two most associated variables was analysed using a multivariable cox regression model. For anti-CCP− individuals, univariable analysis was performed to assess predictors of progression to RA.

## Results

**Fig. 1:**
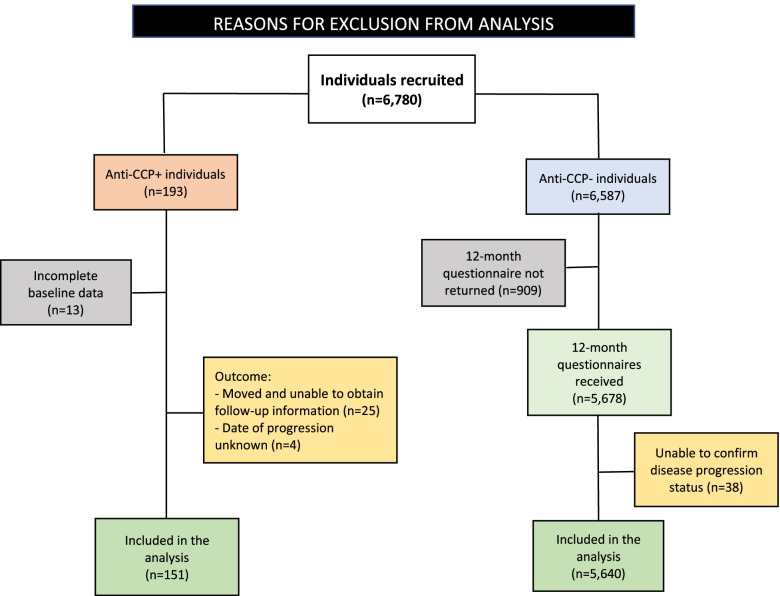
Reasons for exclusion from analysis

A total of 6780 individuals were recruited from 312 primary care practices throughout the UK. Among these, 193 (2.84%) had a positive anti-CCP test and 6587 tested negative (97.15%). The final data set consisted of 151 anti-CCP+ individuals (out of whom 116 physically attended CAH for periodic assessments) and 5640 anti-CCP− subjects. Figure 1 shows reasons for exclusion from the analysis.

### Anti-CCP positive individuals

Mean age was 52 (18–83) years, and the majority were female (62%). Of the 151 anti-CCP+, 65% (98/151) were anti-CCP+ high level and 35% (53/151) were anti-CCP+ low level (Table 1).

**Table 1:** General characteristics of anti-CCP+ low (> 1 to < 3x ULN) and high level (> 3x ULN) individuals (SD = standard deviation)

	Overall anti-CCP+ (*n* = 151)	Anti-CCP+ low level (*n* = 53)	Anti-CCP+ high level (*n* = 98)	*P* value
Sex, female *n* (%)	93 (61.6)	38 (71.7)	55 (56.1)	0.060
Mean age (SD; range) in years	52 (15.2; 19–83)	46 (15.3; 18–77)	55 (14.2; 25–83)	0.001
Mean follow-up (SD; range) in weeks	105 (121.8; 2–560)	133 (117.2; 6–527)	91 (122.1; 2–560)	0.041
Family history of RA, *n* (%)	76 (53.1)	28 (57.1)	48 (51.1)	0.489
Smoking status, *n* (%)	Never 53 (37.1) Ever smoked 90 (62.9)	Never 20 (40.0) Ever smoked 30 (60.0)	Never 33 (35.5) Ever smoked 60 (64.5)	0.590
• Never *n* (%)	53 (37.1)	20 (40.0)	33 (35.5)	
• Previous *n* (%)	65 (45.5)	21 (42.0)	44 (47.3)	
• Current *n* (%)	25 (17.5)	9 (18.0)	16 (17.2)	
Progression to IA, *n* (%)	68 (45%)	7 (13%)	61 (62%)	< 0.001

**Fig. 2:**
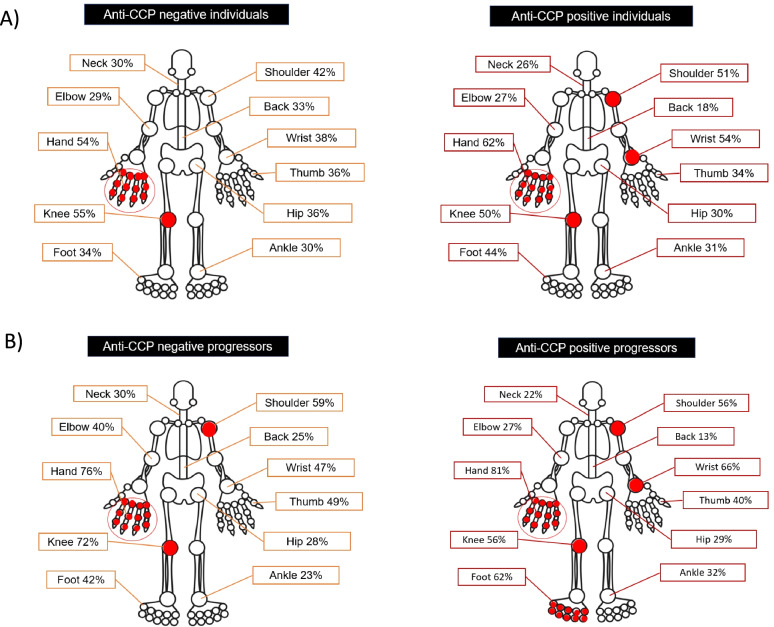
**A** Symptomatic joints at baseline in anti-CCP− and anti-CCP+ individuals. **B** Symptomatic joints at baseline in anti-CCP- and anti-CCP+ individuals who progressed to an IA. Symptomatic joints in > 50% of the subjects are highlighted in red

Half of all anti-CCP+ individuals reported a family history of RA (53%), and most of them (63%) were either previous or current smokers. Forty-five percent of anti-CCP+ individuals (68/151) progressed to IA, and 84% did so in less than 12 months. The mean time of progression was 45 weeks [range 2–494 weeks; median 17 weeks (IQR 8.25–43.00)], and the mean time of follow-up was 105 weeks (range 2–560 weeks). Of the 68 progressors, 63 met the 2010 ACR/EULAR criteria for RA [15], 2 were diagnosed with polymyositis, 2 with undifferentiated IA and 1 with spondyloarthritis. Figure 2 shows the most frequently reported symptomatic joints at baseline.

Subjects were classified into two groups according to their anti-CCP level (Table 1). The majority of low-level individuals were women; their mean age was lower and they had a lower progression rate. There were no significant differences between the groups regarding smoking status and family history of RA; however, smoking exposure was higher among anti-CCP+ high level males (70%) compared with anti-CCP+ high level females (60%). The most striking difference was the proportion of progressors: 62% among the anti-CCP+ high level individuals vs 13% among the low-level ones (*P* < 0.001).

In a multivariable model, high anti-CCP+ level [odds ratio (OR) 9.42; 95% confidence interval (CI) (3.13–28.30), *P* < 0.001], hand pain [OR 2.74; 95% CI (1.03–7.27), *P* = 0.043] and foot pain [OR 4.10; 95% CI (1.59–10.54), *P* = 0.003] were predictive of disease progression (Table 2).

**Table 2:** Baseline predictors for progression to IA in anti-CCP+ individuals

Predictor	Non-progressors (*n* = 83)	Progressors to IA (*n* = 68)	Univariable OR (95% CI) *P*-value	Multivariable OR (95% CI) *P*-value
Mean age (SD; range)	50 (15.65; 18–77)	54 (14.39; 23–83)	1.01 (0.99–1.03) *P* = 0.136	0.95 (0.95–1.02) *P* = 0.504
Female (%)	66	56	0.64 (0.33–1.24) *P* = 0.193	0.56 (0.21–1.45) *P* = 0.234
CCP high level (%)	45	90	**10.83 (4.43–26.48)** ***P*** **< 0.001**	**9.42 (3.13–28.30)** ***P*** **< 0.001**
Family with RA (%)	58	47	0.64 (0.34–1.23) *P* = 0.188	0.56 (0.24–1.34) *P* = 0.198
Ever smoked (%)	55	72	**2.07 (1.04–4.11)** ***P*** **= 0.037**	2.37 (0.96–5.83) *P* = 0.060
Neck (%)	30	22	0.65 (0.72–3.19) *P* = 0.266	0.39 (0.13–1.13) *P* = 0.086
Shoulders (%)	47	56	1.18 (0.13–1.37) *P* = 0.277	0.90 (0.35–2.36) *P* = 0.844
Elbows (%)	28	27	0.93 (0.45–1.93) *P* = 0.865	0.93 (0.33–2.62) *P* = 0.894
Wrists (%)	43	66	**2.55 (1.31–4.96)** ***P*** **= 0.006**	1.28 (0.49–3.36) *P* = 0.607
Hands (%)	47	81	**4.77 (2.27–10.02)** ***P*** **< 0.001**	**2.74 (1.03–7.27)** ***P*** **= 0.043**
Thumbs (%)	29	40	1.61 (0.82–3.19) *P* = 0.164	1.34 (0.44–4.06) *P* = 0.599
Back (%)	23	13	0.51 (0.21–1.22) *P* = 0.133	0.68 (0.20–2.25) *P* = 0.532
Hips (%)	31	29	0.91 (0.45–1.83) *P* = 0.799	1.33 (0.51–3.46) *P* = 0.557
Knees (%)	46	56	1.50 (0.78–2.85) *P* = 0.218	1.08 (0.44–2.69) *P* = 0.855
Ankles (%)	30	32	1.11 (0.55–2.21) *P* = 0.768	0.80 (0.27–2.41) *P* = 0.701
Feet (%)	29	62	**3.97 (2.00–7.85)** ***P*** **< 0.001**	**4.10 (1.59–10.54)** ***P*** **= 0.003**

In fact, absence of hand and foot pain had a negative predictive value (NPV) of 85.4% [95% CI (72.1–92.9), *P* = 0.001] for the development of IA. If the subject also had low anti-CCP+ level, the NPV increased to 95.8 % [95% CI (78.6% to 99.3%), *P* = 0.001]. For individuals with pain in either hands or feet and a high anti-CCP level, the positive predictive value (PPV) was 69.1% [95% CI (63.9% to 73.9%), *P* < 0.001].

**Fig. 3:**
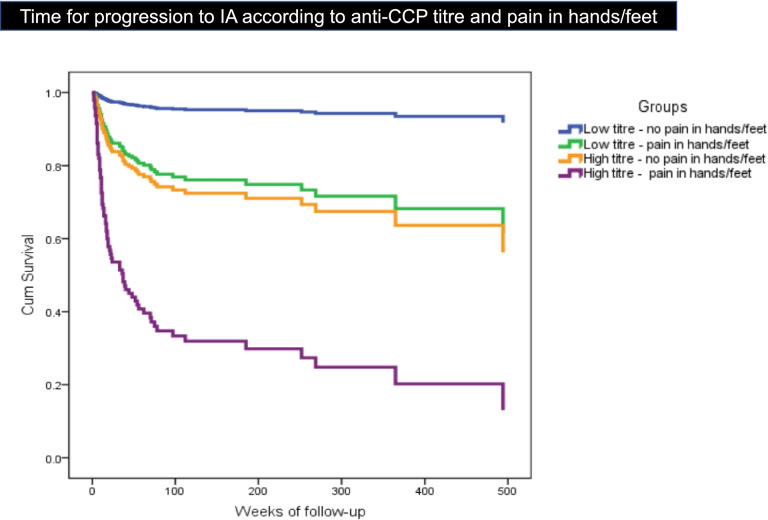
Time for progression to IA according to anti-CCP titre and pain in hands/feet

The rate of progression to IA also varied depending on the presence of pain in hands/feet and the anti-CCP level (Fig. 3). Patients with a low anti-CCP level and no pain in hands/feet had the lowest progression rate, followed by those with low level and pain in hands/feet [hazard ratio (HR) 5.63; 95% CI (0.69–45.95), *P* = 0.107], individuals with a high anti-CCP level but no pain in hands/feet [HR 6.65; 95% CI (0.77–57.13), *P* = 0.084] and finally those with a high anti-CCP level and pain in hands/feet [HR 23.53; 95% CI (3.21–172.34), *P* = 0.002].

Patients without pain in hands/feet (7/68) had a slower progression to IA [mean 125 weeks, (SD 175.01), median 40 weeks (IQR 16–185)] compared with progressors with pain in these joints (61/68) [mean 36.87 weeks (SD 64.29), median 14 weeks (IQR 7.50–38)].

Regardless of the anti-CCP level, all the anti-CCP+ progressors without pain in either hands or feet (7/68) had other additional risk factors: smoking exposure (7/7) and/or family history of RA (6/7).

Additional univariable and multivariable analyses were performed to assess potential association between baseline MSK diagnosis (carpal tunnel syndrome, rotator cuff pathology, trigger finger, tennis elbow and osteoarthritis) and the development of IA; however, none of them was statistically significant (Supplementary Table 1).

Finally, there were no differences in the proportion of progressors between the anti-CCP+ participants who were regularly attending clinic at CAH and those who remained under GP care [43% (50/116) and 51% (18/35) of progressors respectively, *P* = 0.386].

### Anti-CCP negative individuals

Mean age of anti-CCP negative individuals was 52 (16–91, SD 14.7) years, and 72% were female. Thirty eight percent reported having a FDR with RA and 38% were either current or former smokers. A total of 5678 individuals returned their 12-month questionnaires, of whom 239 reported progression to IA (4.2%). The disease status of 38/239 individuals could not be confirmed by a GP; therefore, only 201/239 were included in the analysis. Of these 201, GPs discounted IA in 148 participants and confirmed IA in 53, representing 0.93% (53/5640) of progressors among all anti-CCP negative individuals.

Twenty one of these 53 progressors were diagnosed with RA; 13 with spondyloarthritis, 11 with polymyalgia rheumatica (requiring DMARDs for joint swelling), 3 with polymyositis, 3 with systemic lupus erythematosus and 2 with systemic sclerosis. Only progression (yes/no) to IA within the first 12 months was recorded, and therefore, no data are available regarding the mean time of progression.

Figure 2 shows the most symptomatic joints at baseline. Progressors had a higher percentage of symptomatic joints and a higher number of other MSK diagnoses such as carpal tunnel syndrome (CTS), rotator cuff pathology and osteoarthritis (Supplementary Table 2).

Multivariable analysis (Table 3) showed that hand [OR 2.17; 95% CI (1.17–5.39), *P* = 0.018] and knee pain [OR 2.65; 95% CI (1.47–6.25), *P* = 0.003] were associated with the development of IA within the following 12 months. Older age showed only a slightly higher risk for IA [OR 1.04; 95% CI (1.02–1.07), *P* < 0.001].

**Table 3:** Baseline predictors for progression to IA in anti-CCP− individuals

Predictor	Non-progressors (*n* = 5587)	Progressors to IA (*n* = 53)	Univariable OR (95% CI) *P*-value	Multivariable OR (95% CI) *P*-value
Mean age (SD; range)	53 (14.7; 16–91)	60 (13.62; 30–82)	**1.04 (1.02–1.06)** ***P*** **< 0.001**	**1.04 (1.02–1.07)** ***P*** **< 0.001**
Female (%)	72	58	**0.54 (0.31–0.94)** ***P*** **= 0.030**	0.67 (0.35–1.28) *P* = 0.229
Family with RA (%)	38	33	0.758 (0.42–1.35) *P* = 0.349	1.14 (0.59–2.19) *P* = 0.683
Ever smoked (%)	38	43	1.25 (0.69–2.28) *P* = 0.462	1.11 (0.60–2.05) *P* = 0.730
Neck (%)	30	29	1.03 (0.57–1.8) *P* = 0.932	0.62 (0.29–1.30) *P* = 0.202
Shoulders (%)	41	59	**1.99 (1.15–3.45)** ***P*** **= 0.014**	1.95 (0.98–3.86) *P* = 0.056
Elbows (%)	29	40	1.58 (0.91–2.75) *P* = 0.104	1.32 (0.67–2.59) *P* = 0.413
Wrists (%)	38	47	1.43 (0.83–2.46) *P* = 0.193	1.18 (0.60–2.31) *P* = 0.625
Hands (%)	53	76	**2.67 (1.43–5.02)** ***P*** **= 0.002**	**2.51 (1.17–5.39)** ***P*** **= 0.018**
Thumbs (%)	36	49	1.70 (0.99–2.92) *P* = 0.054	1.23 (0.63–2.41) *P* = 0.541
Back (%)	33	25	0.66 (0.35–1.23) *P* = 0.190	0.66 (0.31–1.41) *P* = 0.279
Hips (%)	36	28	0.69 (0.38–1.26) *P* = 0.226	0.57 (0.28–1.17) *P* = 0.127
Knees (%)	55	72	**2.07 (1.14–3.77)** ***P*** **= 0.018**	**3.03 (1.47–6.25)** ***P*** **= 0.003**
Ankles (%)	30	23	0.68 (0.36–1.29) *P* = 0.239	0.55 (0.25–1.21) *P* = 0.140
Feet (%)	34	42	1.37 (0.79–2.38) *P* = 0.256	1.04 (0.52–2.06) *P* = 0.910

RA was the most frequent diagnosis among the anti-CCP− progressors, and univariable analysis showed that pain in hands [OR 5.21; 95% CI (1.53–7.69), *P* = 0.008], thumbs [OR 2.87; 95% CI (1.19–6.93), *P* = 0.019], older age [OR 1.04; 95% CI (1.01–1.07), *P* = 0.026] and CTS [OR 3.49; 95% CI (1.40–8.67), *P* = 0.007] were associated with a higher risk of progression to RA (Supplementary Table 3). Multivariable analysis could not be performed to assess predictors of RA in anti-CCP− individuals due to the low number of patients per variable.

**Fig. 4:**
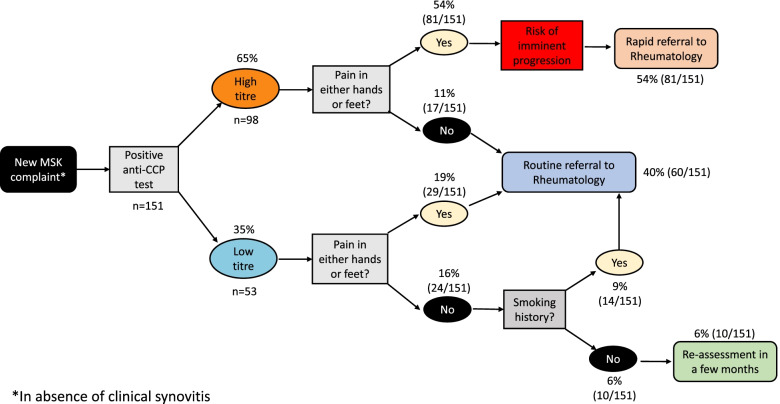
Proposed primary care anti-CCP positive pathway

Based on the results of the study, Fig. 4 has been elaborated to provide clear guidance for primary care physicians attending patients with a new non-specific MSK complaint, who test positive for anti-CCP antibodies.

## Discussion

MSK complaints account for 30% of GP consultations in England [16]. However, in the setting of an open access service to rheumatology, it can be difficult to manage the large number of referrals of individuals with MSK symptoms without clinical synovitis; even if these are restricted to the anti-CCP+ ones. Considering this, there is need for prioritisation, and this study provides practical guidance for primary care physicians to easily assess the urgency of referral to rheumatology using widely available tests. This has become especially relevant with the COVID19-SARS2 pandemic, which resulted in rheumatology appointment cancellations and a significant increase in the waiting times for specialist assessment. Considering this, one of the advantages of the proposed assessment is the possibility of remote performance if required.

To our knowledge, this is the largest reported prospective primary care study of individuals at-risk of RA. Its approach stands out from other studies due to the significant role that patient reported factors (especially symptoms) play in assessing the risk of progression and its simplicity and feasibility for use in primary care.

Our study has confirmed the enriched prevalence of anti-CCP antibodies in individuals with a new non-specific MSK complaint (2.84%) in a larger cohort, as well as the risk of rapid disease progression, with 45% being diagnosed with IA with a mean time to progression of 45 weeks, as Nam et al. reported [11]. Results from secondary care are heterogeneous: whereas Rakieh et al. [17] also had a high number of progressors (50% of anti-CCP+ subjects with non-specific MSK complaints), two Dutch studies [18, 19] reported 20% and 35% progressors respectively among seropositive patients with arthralgia. The latter studies included patients with either rheumatoid factor (RF) or anti-CCP antibodies; the fact that RF is less specific for disease progression in the at-risk phase [18, 20] and the different settings (specialist assessments in patients already referred) could explain the discrepancy.

In our study, subjects were analysed in groups according to anti-CCP positivity/negativity but also the anti-CCP level. We found an association between the anti-CCP level and the development of IA, with 62% of high-level individuals developing IA. This association had also been suggested by a retrospective study with anti-CCP+ individuals without RA [21], reporting a progression rate of 46% among those with a high level. However, 76% of their individuals had arthralgia and they were all recruited in tertiary care. These results differ from another study [22], which did not find any correlation between the anti-CCP level and progression to IA; but only 13% of the individuals in the cohort were anti-CCP+.

In our cohort, the majority of subjects from both anti-CCP low and high-level groups were female. This is consistent with a study that reported association between female sex and anti-CCP positivity [10]. Even though the incidence of RA is higher in women [23], we did not find female sex to be predictive of progression in either anti-CCP+ or anti-CCP− individuals. A higher percentage of men were anti-CCP+ high-level, perhaps due to the increased smoking exposure in males in the current study [24].

Smoking has been associated not only with the development of anti-CCP antibodies but also with development of anti-CCP+ RA [21, 25]. The results of the univariable analysis in our anti-CCP+ individuals were in line with this. Multivariable analysis did not show strict association between smoking exposure and development of IA; however, this could be due to the low number of participants, as it nearly reached significance (*P* = 0.06). In addition, all the anti-CCP+ progressors that did not have pain in hands/feet, had smoking exposure as a risk factor. A previous study found that smoking increased the risk of developing anti-CCP negative RA, but this risk disappeared 20 years after smoking cessation [25]. We did not find any correlation between smoking exposure and development of IA for anti-CCP− patients, but we did not assess the time passed since smoking cessation.

It is known that RA has a predilection for targeting small joints [15]; for anti-CCP+ subjects, hands and feet seem to be a key symptomatic area as 92% of the anti-CCP+ high level progressors reported hand and/or foot pain. In addition, all anti-CCP+ low level progressors except one presented with hand or foot pain. As expected, these regions showed association with the development of IA. Most importantly, the high NPV of absence of pain in hands or feet combined with a low anti-CCP level (> 95%) should reassure clinicians that such an individual is unlikely to develop IA at that point, and therefore, referral to rheumatology is not a priority (nevertheless, this should not discourage referral if a rheumatic disease is suspected) (Fig. 4).

In addition, the classification of subjects in four groups based on the anti-CCP level and “presence/absence of pain in hands or feet” can be useful to estimate the urgency of specialist assessment (Fig. 3).

Surprisingly, wrist pain (in anti-CCP+ and anti-CCP− individuals) and foot pain (in anti-CCP− individuals) were not associated with disease progression. For wrist pain, this could be explained by some subjects reporting it as hand pain. As far as foot pain is concerned, a recent study compared the radiological pattern of seronegative and seropositive RA: they reported significant differences, not only in the degree of damage but also in the joint distribution [26]. Whereas in seropositive RA most erosions occurred in the feet, these joints seemed to be spared in seronegative RA.

Data interpretation is more complex for anti-CCP− individuals due to the heterogenicity of the progressors’ IA diagnoses. Whereas 93% of the anti-CCP+ progressors met the ACR/EULAR 2010 criteria for RA, only 40% of the anti-CCP− progressors did. Even though a retrospective primary care study also found association between knee and/or hand pain and progression [27], pain in these sites should be seen as a warning sign in anti-CCP− subjects and the differential diagnosis should be guided by the clinical picture of the patient, bearing in mind other risk factors (e.g. early morning stiffness in suspected RA).

One of the main strengths of this study is the number of participants and the prospective data collection. The fact that individuals were recruited in hundreds of primary care centres across the UK means that our results should be representative of the wider background population. Additionally, it is also the first study to prospectively investigate the outcome of MSK symptoms in a large anti-CCP negative cohort.

One limitation is the fact that anti-CCP status was not re-checked at the moment of progression in anti-CCP− participants. However, studies suggest that anti-CCP antibodies appear in low levels several years before RA diagnosis and they only increase 2–4 years before IA development [28]. Considering this, it is unlikely that the anti-CCP status of the anti-CCP− patients could have changed in only 12 months. This shorter follow-up could have contributed to the low number of anti-CCP− progressors and is actually a limitation itself: whereas anti-CCP+ patients were followed-up for over 10 years, the follow-up of anti-CCP− patients was restricted to 12 months, which means that there is potential progression data that were not collected.

It is possible that there could be a bias regarding a more likely IA diagnosis in anti-CCP+ individuals compared with anti-CCP− participants, as the majority of the former group were attending clinic regularly. However, data was compared between anti-CCP+ participants attending clinic and anti-CCP+ individuals who continued under GP care, and no significant differences were observed; therefore, it is unlikely that IA in anti-CCP− individuals was underdiagnosed.

Another limitation is the missing data, and in contrast with the IA status (whose diagnosis by a rheumatologist was confirmed by the GP), epidemiological information was self-reported by the participants. When asked about family history of RA, subjects often have trouble differentiating between RA and osteoarthritis. This could have led to an overestimation of individuals with RA relatives that could explain why association between first-degree relative with RA and disease progression was not found. In addition, this confusion between types of arthritis could also explain why progression to IA was overreported in the questionnaires: the diagnosis of IA could only be confirmed in 26% of subjects that self-reported progression. These examples indicate that a patient reported diagnosis of rheumatic diseases is not completely reliable and the distribution of joint pain seems to be a more useful tool when assessing the need for referral to specialist rheumatology services.

## Conclusion

The increased demand for access to specialist rheumatology services (exacerbated post COVID19-SARS2 pandemic) reinforces the need for a prioritisation model for patients with new MSK symptoms. This study shows that risk stratification can be achieved using tests available in primary care, in combination with patient reported joint symptoms.

Our results indicate that individuals without clinical synovitis who have pain in the hands/feet and a high anti-CCP level are likely to have a rapid disease progression. In contrast, low level anti-CCP+ individuals without clinical synovitis who do not have pain in hands/feet are very unlikely to progress to IA. While in anti-CCP− individuals the risk of progression is low, hand and knee pain may be seen as a red flag that requires follow-up. This provides useful discrimination that may be used to prioritise referrals to rheumatology and avoid diagnostic delay.

## Supplementary Information

**Additional file 1: Supplementary Table 1.** Musculoskeletal conditions at baseline and their association with IA development in anti-CCP+ individuals. (Multivariable analysis has been adjusted for confounders: age, gender, anti-CCP titre, first degree relative with RA and smoking history).

**Additional file 2: Supplementary Table 2.** Musculoskeletal conditions at baseline and their association with developing an IA in anti-CCP− individuals. Multivariable analysis has been adjusted for confounders (age, gender, first degree relative with RA and smoking history).

**Additional file 3: Supplementary Table 3.** Baseline predictors for progression to rheumatoid arthritis in anti-CCP− individuals. Univariable analysis.

## Data Availability

The datasets used and/or analysed during the current study are available from the corresponding author on reasonable request.

## Abbreviations

ACPA: Anti-cyclic citrullinated peptide antibodies
Anti-CCP+/−: Anti-cyclic citrullinated peptide positive/negative
CAH: Chapel Allerton Hospital
CI: Confidence interval
CRN: Clinical Research Network
CTS: Carpal tunnel syndrome
DMARDs: Disease-modifying antirheumatic drugs
GP: General practitioner
HR: Hazard ratio
IA: Inflammatory arthritis
IQR: Interquartile range
MSK: Musculoskeletal
NHIR: National Institute of Health Research
NPV: Negative predictive value
OR: Odds ratio
PPV: Positive predictive value
RA: Rheumatoid arthritis
SD: Standard deviation
UK: United Kingdom
US: Ultrasound

## References

[1] Emery P, Breedveld FC, Dougados M, Kalden JR, Schiff MH, Smolen JS (2002). Early referral recommendation for newly diagnosed rheumatoid arthritis: evidence based development of a clinical guide. Ann Rheum Dis.

[2] Gwinnutt JM, Symmons DPM, MacGregor AJ, Chipping JR, Marshall T, Lunt M (2017). Twenty-year outcome and association between early treatment and mortality and disability in an inception cohort of patients with rheumatoid arthritis: results from the Norfolk Arthritis Register. Arthritis Rheum.

[3] National Audit Office (2009). Services for people with rheumatoid arthritis.

[4] Guide to NHS waiting times in England. https://www.nhs.uk/using-the-nhs/nhs-services/hospitals/guide-to-nhs-waiting-times-in-england/#maximum. Accessed 30 Sep 2020.

[5] Mankia K, Di Matteo A, Emery P. Prevention and cure: the major unmet needs in the management of rheumatoid arthritis. J Autoimmun. 2020;110:102399. 10.1016/j.jaut.2019.102399 31899021

[6] Raza K, Breese M, Nightingale P, Kumar K, Potter T, Carruthers DM (2005). Predictive value of antibodies to cyclic citrullinated peptide in patients with very early inflammatory arthritis. J Rheumatol.

[7] van Gaalen FA, Linn-Rasker SP, van Venrooij WJ, de Jong BA, Breedveld FC, Verweij CL (2004). Autoantibodies to cyclic citrullinated peptides predict progression to rheumatoid arthritis in patients with undifferentiated arthritis: a prospective cohort study. Arthritis Rheum.

[8] Rantapaa-Dahlqvist S, de Jong BA, Berglin E, Hallmans G, Wadell G, Stenlund H (2003). Antibodies against cyclic citrullinated peptide and IgA rheumatoid factor predict the development of rheumatoid arthritis. Arthritis Rheum.

[9] Tasliyurt T, Kisacik B, Kaya SU, Yildirim B, Pehlivan Y, Kutluturk F (2013). The frequency of antibodies against cyclic citrullinated peptides and rheumatoid factor in healthy population: a field study of rheumatoid arthritis from northern Turkey. Rheumatol Int.

[10] van Zanten A, Arends S, Roozendaal C, Limburg PC, Maas F, Trouw LA (2017). Presence of anticitrullinated protein antibodies in a large population-based cohort from the Netherlands. Ann Rheum Dis.

[11] Nam JL, Hunt L, Hensor EM, Emery P (2016). Enriching case selection for imminent RA: the use of anti-CCP antibodies in individuals with new non-specific musculoskeletal symptoms - a cohort study. Ann Rheum Dis.

[12] Hider SL, Muller S, Helliwell T, Prior JA, Scott I, Lawton SA (2019). Symptoms associated with inflammatory arthritis are common in the primary care population: results from the joint symptoms survey. Rheumatology (Oxford, England).

[13] Muller S, Hider S, Machin A, Stack R, Hayward RA, Raza K (2019). Searching for a prodrome for rheumatoid arthritis in the primary care record: A case-control study in the clinical practice research datalink. Semin Arthritis Rheum.

[14] Almoallim H, Janoudi N, Attar SM, Garout M, Algohary S, Siddiqui MI (2017). Determining early referral criteria for patients with suspected inflammatory arthritis presenting to primary care physicians: a cross-sectional study. Open Access Rheumatol: Res Rev.

[15] Aletaha D, Neogi T, Silman AJ, Funovits J, Felson DT, Bingham CO (2010). 2010 rheumatoid arthritis classification criteria: an American College of Rheumatology/European League Against Rheumatism collaborative initiative. Ann Rheum Dis.

[16] NHS England. Elective Care Transformation Programme: helping people with painful bone and joint conditions see the right person. https://www.englandnhsuk/elective-care-transformation/best-practice-solutions/musculoskeletal/. Accessed 16 Feb 2021.

[17] Rakieh C, Nam JL, Hunt L, Hensor EM, Das S, Bissell LA (2015). Predicting the development of clinical arthritis in anti-CCP positive individuals with non-specific musculoskeletal symptoms: a prospective observational cohort study. Ann Rheum Dis.

[18] Bos WH, Wolbink GJ, Boers M, Tijhuis GJ, de Vries N, van der Horst-Bruinsma IE (2010). Arthritis development in patients with arthralgia is strongly associated with anti-citrullinated protein antibody status: a prospective cohort study. Ann Rheum Dis.

[19] van de Stadt LA, Witte BI, Bos WH, van Schaardenburg D (2013). A prediction rule for the development of arthritis in seropositive arthralgia patients. Ann Rheum Dis.

[20] van Steenbergen HW, Mangnus L, Reijnierse M, Huizinga TW, van der Helm-van Mil AH (2016). Clinical factors, anticitrullinated peptide antibodies and MRI-detected subclinical inflammation in relation to progression from clinically suspect arthralgia to arthritis. Ann Rheum Dis.

[21] Ford JA, Liu X, Marshall AA, Zaccardelli A, Prado MG, Wiyarand C, et al. Impact of cyclic citrullinated peptide antibody level on progression to rheumatoid arthritis in clinically tested CCP-positive patients without RA. Arthritis Care Res. 2019;71(12):1583-92. 10.1002/acr.23820 PMC6586539 30570827

[22] Ten Brinck RM, van Steenbergen HW, van Delft MAM, Verheul MK, Toes REM, Trouw LA (2017). The risk of individual autoantibodies, autoantibody combinations and levels for arthritis development in clinically suspect arthralgia. Rheumatology (Oxford, England).

[23] Brennan P, Silman A (1995). Why the gender difference in susceptibility to rheumatoid arthritis?. Ann Rheum Dis.

[24] Ishikawa Y, Ikari K, Hashimoto M, Ohmura K, Tanaka M, Ito H (2019). Shared epitope defines distinct associations of cigarette smoking with levels of anticitrullinated protein antibody and rheumatoid factor. Ann Rheum Dis.

[25] Hedstrom AK, Stawiarz L, Klareskog L, Alfredsson L (2018). Smoking and susceptibility to rheumatoid arthritis in a Swedish population-based case-control study. Eur J Epidemiol.

[26] Gadeholt O, Hausotter K, Eberle H, Klink T, Pfeil A (2019). Differing X-ray patterns in seronegative and seropositive rheumatoid arthritis. Clin Rheumatol.

[27] Beers-Tas MV, Nielen MM, Twisk JWR, Korevaar J, van Schaardenburg D. Increased primary care use for musculoskeletal symptoms, infections and comorbidities in the years before the diagnosis of inflammatory arthritis. RMD Open. 2020;6(2):1-9. 10.1136/rmdopen-2019-001163 PMC7425115 32641448

[28] van de Stadt LA, de Koning MH, van de Stadt RJ, Wolbink G, Dijkmans BA, Hamann D (2011). Development of the anti-citrullinated protein antibody repertoire prior to the onset of rheumatoid arthritis. Arthritis Rheum.

