# Supplementary material for: Prioritising referrals of individuals at-risk of RA: guidance based on results of a 10-year national primary care observational study
Source: Arthritis Res Ther. 2022 Jan 18;24:26. doi: 10.1186/s13075-022-02717-w (PMC8767684; doi:10.1186/s13075-022-02717-w)
Supplement: Supplementary file 1 — Additional file 1: Supplementary Table 1. Musculoskeletal conditions at baseline and their association with IA development in anti-CCP+ individuals. (Multivariable analysis has been adjusted for confounders: age, gender, anti-CCP titre, first degree relative with RA and smoking history). [file 13075_2022_2717_MOESM1_ESM.docx]

| PREDICTOR | Non-progressors (n=83) | Progressors to  IA (n=68) | Univariable  OR (95% CI) P-value | Multivariable  OR (95% CI) P-value |
| --- | --- | --- | --- | --- |
| **Mean age (range)** | 50  (18-77) | 54  (23-83) | 1.01 (0.99-1.03) P=0.136 | 1.01 (0.98-1.04)  P=0.633 |
| **Female (%)** | 66 | 56 | 0.64 (0.33-1.24) P=0.193 | 0.76 (0.31-1.86)  P=0.541 |
| **CCP high titre (%)** | 45 | 90 | **10.83 (4.43-26.48) P<0.001** | **16 (5.34-48.30)**  **P<0.001** |
| **Family with RA (%)** | 58 | 47 | 0.64 (0.34-1.23) P=0.188 | 1.85 (0.80-4.26)  P=0.147 |
| **Ever smoked (%)** | 55 | 72 | **2.07 (1.04-4.11) P=0.037** | 0.49 (0.20-1.17)  P=0.108 |
| **Carpal tunnel syndrome (%)** | 7 | 5 | 1.54 (0.35-6.72)  P=0.564 | 1.08 (0.16-7.20)  P=0.938 |
| **Rotator cuff (%)** | 12 | 12 | 1.02 (0.37-2.82)  P=0.973 | 1.45 (0.36-5.77)  P=0.599 |
| **Trigger finger (%)** | 0 | 5 | --- | --- |
| **Tennis elbow (%)** | 12 | 11 | 1.18 (0.41-3.38)  P=0.753 | 0.81 (0.19-3.51)  P=0.782 |
| **Osteoarthritis (%)** | 21 | 14 | 1.64 (0.66-4.05)  P=0.286 | 2.92 (0.87-9.75)  P=0.082 |

**Supplementary table 1**. Musculoskeletal conditions at baseline and their association with IA development in anti-CCP+ individuals. (Multivariable analysis has been adjusted for confounders: age, sex, anti-CCP titre, first degree relative with RA and smoking history).
