# Supplementary material for: Prioritising referrals of individuals at-risk of RA: guidance based on results of a 10-year national primary care observational study
Source: Arthritis Res Ther. 2022 Jan 18;24:26. doi: 10.1186/s13075-022-02717-w (PMC8767684; doi:10.1186/s13075-022-02717-w)
Supplement: Supplementary file 2 — Additional file 2: Supplementary Table 2. Musculoskeletal conditions at baseline and their association with developing an IA in anti-CCP− individuals. Multivariable analysis has been adjusted for confounders (age, gender, first degree relative with RA and smoking history). [file 13075_2022_2717_MOESM2_ESM.docx]

| PREDICTOR | Non-progressors  (n=5587) | Progressors to IA (n=53) | Univariable  OR (95% CI) P-value | Multivariable  OR (95% CI) P-value |
| --- | --- | --- | --- | --- |
| Mean age (range) | 53 (16-91) | 60 (30-82) | **1.04 (1.02-1.06) P<0.001** | **1.04 (1.02-1.07) P<0.001** |
| Female (%) | 72 | 58 | **0.54 (0.31-0.94) P=0.030** | 0.67 (0.35-1.28) P=0.229 |
| Family with RA (%) | 38 | 33 | 0.758 (0.42-1.35) P=0.349 | 1.14 (0.59-2.19) P=0.683 |
| Ever smoked (%) | 38 | 43 | 1.25 (0.69-2.28) P=0.462 | 1.11 (0.60-2.05) P=0.730 |
| Carpal tunnel syndrome (%) | 13 | 17 | 1.62 (0.81-3.24) P=0.172 | 0.56 (0.25-1.26)  P=0.162 |
| Rotator cuff (%) | 12 | 19 | 1.77 (0.88-3.53) P=0.108 | 0.47 (0.22-1.02)  P=0.056 |
| Trigger finger (%) | 5 | 4 | 1.20 (0.37-3.88) P=0.758 | 1.35 (0.30-5.97)  P=0.696 |
| Tennis elbow (%) | 15 | 10 | 0.73 (0.31-1.70) P=0.460 | 2.22 (0.77-6.35)  P=0.139 |
| Osteoarthritis (%) | 18 | 25 | 1.69 (0.91-3.12) P=0.096 | 0.79 (0.38-1.62)  P=0.519 |

**Supplementary table 2**. Musculoskeletal conditions at baseline and their association with developing an IA in anti-CCP- individuals. Multivariable analysis has been adjusted for confounders (age, sex, first degree relative with RA and smoking history).
