# Supplementary material for: Prioritising referrals of individuals at-risk of RA: guidance based on results of a 10-year national primary care observational study
Source: Arthritis Res Ther. 2022 Jan 18;24:26. doi: 10.1186/s13075-022-02717-w (PMC8767684; doi:10.1186/s13075-022-02717-w)
Supplement: Supplementary file 3 — Additional file 3: Supplementary Table 3. Baseline predictors for progression to rheumatoid arthritis in anti-CCP− individuals. Univariable analysis. [file 13075_2022_2717_MOESM3_ESM.docx]

| PREDICTOR | RA progressors  (n=21) | Non-progressors  (n=5619) | Predictors for RA Univariable  OR (95% CI) P-value |
| --- | --- | --- | --- |
| Mean age in years (range) | **60**  **(30-82)** | **53**  **(16-91)** | **1.04 (1.01-1.07) p=0.026** |
| Female (%) | 57 | 72 | 0.52 (0.22-1.23) p=0.14 |
| Family with RA (%) | 33 | 38 | 0.80 (0.32-1.20) p=0.639 |
| Ever smoked (%) | 40 | 38 | 1.10 (0.39-3.09) p=0.862 |
| Neck (%) | 19 | 30 | 0.56 (0.19-1.66) p=0.293 |
| Shoulders (%) | 57 | 41 | 1.88 (0.79-4.46) p=0.154 |
| Elbows (%) | 33 | 29 | 1.2 (0.48-2.98) p=0.692 |
| Wrists (%) | 52 | 38 | 1.76 (0.75-4.16) p=0.195 |
| Hands (%) | 86 | **54** | **5.21 (1.53-7.69) p=0.008** |
| Thumbs (%) | 62 | **36** | **2.87 (1.19-6.93) p=0.019** |
| Back (%) | 24 | 33 | 0.63 (0.23-1.73) p=0.373 |
| Hips (%) | 19 | 36 | 0.41 (0.14-1.23) p=0.111 |
| Knees (%) | 62 | 55 | 1.32 (0.55-3.19) p=0.537 |
| Ankles (%) | 24 | 30 | 0.73 (0.27-1.99) p=0.533 |
| Feet (%) | 38 | 34 | 1.19 (0.49-2.88) p=0.700 |
| Carpal tunnel syndrome (%) | 33 | **13** | **3.49 (1.40-8.67) p=0.007** |
| Rotator cuff (%) | 19 | 12 | 1.78 (0.60-5.31) p=0.301 |
| Trigger finger (%) | 5 | 5 | 1.00 (0.13-7.48) p=1.000 |
| Tennis elbow (%) | 10 | 15 | 0.60 (0.14-2.58) p=0.491 |
| Osteoarthritis (%) | 24 | 18 | 1.46 (0.53-4.00) p=0.460 |

**Supplementary table 3**. Baseline predictors for progression to rheumatoid arthritis in anti-CCP- individuals. Univariable analysis.
